# Impact of guided self-study on learning success in undergraduate physiotherapy students in Switzerland – a feasibility study of a higher education intervention

**DOI:** 10.1186/s12909-021-02794-6

**Published:** 2021-06-29

**Authors:** Slavko Rogan, Jan Taeymans, Stefan Zuber, Evert Zinzen

**Affiliations:** 1 Bern University of Applied Sciences, Department of Health Professions, Division of Physiotherapy, Bern, Switzerland; 2 Academy for integrative physiotherapy and training education, Grenzach-Wyhlen, Germany; 3 Vrije Universiteit Brussel, Faculty of Physical Education and Physiotherapy, Brussels, Belgium

**Keywords:** Higher education, Learning success, Self-study

## Abstract

**Background:**

Guided self-study (G-SS) can be used as a self-directed learning method or self-determined learning that fosters changes in knowledge and skills in a higher physiotherapy education setting. Until now, there has been no empirical evidence for the use of G-SS in higher physiotherapy education. This study aimed to investigate the feasibility to establish a G-SS program in a fulltime undergraduate physiotherapy degree course. In addition, the effectiveness of the G-SS was assessed on changes in knowledge and skills.

**Method:**

Fifty-one first-semester physiotherapy students were randomly divided into a G-SS group or control group (CG). The G-SS group received six clinical cases. Each case was processed in an eight-day cycle. One week in advance, the clinical case were provided to the students electronically (day 1). The students prepared the cases in groups and were guided by the tutor during this preparation time (day 2 to 7). Group work results were presented and reflected on during a moderated plenum session at day 8. A priori criteria of success were defined based on empirical experience for the primary outcome parameters i) exposure, ii) responsiveness of students and iii) program differentiation. The secondary outcome was the total score in the objective structured clinical examination (OSCE) and written exams. Statistical analyses were conducted using SPSS.

**Results:**

The responsiveness of students as willing to participate in the G-SS program was 23%, clearly below the a priori set 83%. No differences in program differentiation were found. G-SS as compared to the CG scored significantly better on OSCE (*p* = 0.003) and on the written exam (p = 0.004).

**Conclusion:**

The results showed that this higher education G-SS program in its current form was not feasible. Slight modification of the study protocol (e.g. better time planning in the academic calendar) is needed to improve the student’s responsiveness. The adjustments to the timetable must allow the physiotherapy students to prepare the clinical cases under conditions of lower workload. G-SS has the potential to promote change in knowledge and skills in undergraduate physiotherapy students when students prepare and present the clinical case solutions and reflect upon their actions.

**Trial registration:**

Registry of Efficacy and Effectiveness Studies, Registry ID: #1726.1 Registered on February 26th, 2019.

## Background

The shift away from traditional concepts of work requires individuals who can adapt quickly to new situations and know how to acquire knowledge. Within this changing world, humans should learn more independently, in preparation for higher education, work and life [1]. To prepare humans for changes in the world of work, pedagogical methods are no longer fully sufficient [2]. Previously, learning was related to childhood, but lifelong independent learning is becoming increasingly essential.

The higher education landscape must satisfy this requirement with the implementation of adult education. This includes andragogy which focusses on self-directed learning (SDL) or heutagogy which focusses on self-determined learning (SDtL).

Knowles [3] defined andragogy as the art and science of helping adults learning, in contrast to pedagogy which has been defined as the art and science of teaching children. The use of clinical cases may be an example of andragogy on an undergraduate level in the health professions domain. The higher education teacher defines the learning outcome and becomes a facilitator, who supports the adult learners and diagnoses individual learning needs. As a facilitator, the higher education teacher plans active sequences, choosing the most effective teaching and evaluation methods.

Heutagogy depicts self-determined learning as a holistic, and lifelong process [4]. The learner is in the center and define the learning outcome [5]. For example, the learner develops a clinical case on graduate or doctoral level. The higher education teacher’s emphasis changes and teacher-centered instruction (TCI) is avoided. The method of heutagogy has not yet established in the German-speaking countries.

Based on the Bologna Process, the Rektorenkonferenz der Fachhochschulen der Schweiz [6] defined that academic programs in Switzerland are divided into required time of attendance session (e.g. TCI, workshops, seminars) and self-study (free self-study (F-SS) and guided self-study (G-SS)). In the context of higher education self-study could be used to foster SDL and SDtL.

### Theoretical background and the formulation of research question

Self-study is an essential component of scientific higher education [7, 8]. Two forms of self-study have been proposed: F-SS and G-SS [8, 9]. While students are self-reliant in F-SS, they will be supported by lecturers or tutors during G-SS [9]. Both self-study forms are implemented in the higher physiotherapy education curricula at the Bern University Applied of Science (BFH). In the context of learning practical physiotherapy skills in higher education, understanding and applying the mechanisms of G-SS is of particular importance. Up to now, there has been no standardized procedure for G-SS at the BFH. Based on information provided by Landwehr and Mueller [8] and Rogan [9], a standardized procedure to implement G-SS was established.

Landwehr and Mueller postulated five phases for G-SS [8]. During a preparatory phase (phase one), students receive a learning assignment (clinical case description) from the tutor with clear-cut learning objectives. Phase two consists of the realization stages one and two in which the students work independently on the learning assignment. The tutor provides coaching (stage one) and checking (stage two) to the students. During phase three the students present an insight into their learning outcomes to the tutor and their fellow students (e.g. during a plenum session). Both students and tutor will reflect on the learning process during phase four. Finally, in phase five, the students give each other peer feedback on their presentations and learning processes. Rogan [9] proposed a theoretical model on the structure of G-SS and on how physiotherapy students gain knowledge in practical physiotherapy skills. However, empirical evidence of this theoretical model according to Rogan [9] is still lacking.

Therefore, an empirical study to gain evidence should be planned. As a first step in a line of research, the feasibility and acceptability of a planned study should be investigated as a feasibility study [10]. A feasibility study also includes the examination of intervention impact, but this is a secondary goal [10, 11].

It is known that in medical education evidence was generated on prejudices, hunches, opinions, and guesses [12]. As in other disciplines, deficiencies in medical education have been identified for reporting quality of published studies [13] and the lack of robust study designs [14]. This study used the Kirkpatrick’s four level evaluation model [15] including 1. learners’ satisfaction, 2. changes in knowledge and skills, 3. changes in behavior and 4. changes to the patient outcome behavior and results to evaluate the learning effectiveness of the guided self-study intervention model of the BFH. The Kirkpatrick’s four level evaluation model is employed widely in education research [16].

This research project will investigate the feasibility (primary aim) and the potential impact (secondary aim) of G-SS on changes in knowledge and skills based on Rogan’s theoretical work [9], into one cohort of an undergraduate physiotherapy higher education fulltime program at BFH, Switzerland. The primary aim of this feasibility study was to report the fidelity of implementation of the G-SS. A secondary aim was to identify changes in knowledge and skills of the learners (i.e. impact of the G-SS). The research question in this present feasibility study was: is it possible to develop a study to evaluate changes in knowledge and skills in undergraduate physiotherapy students?

## Methods

### Study design, setting, quality reporting, ethics

**Fig. 1:**
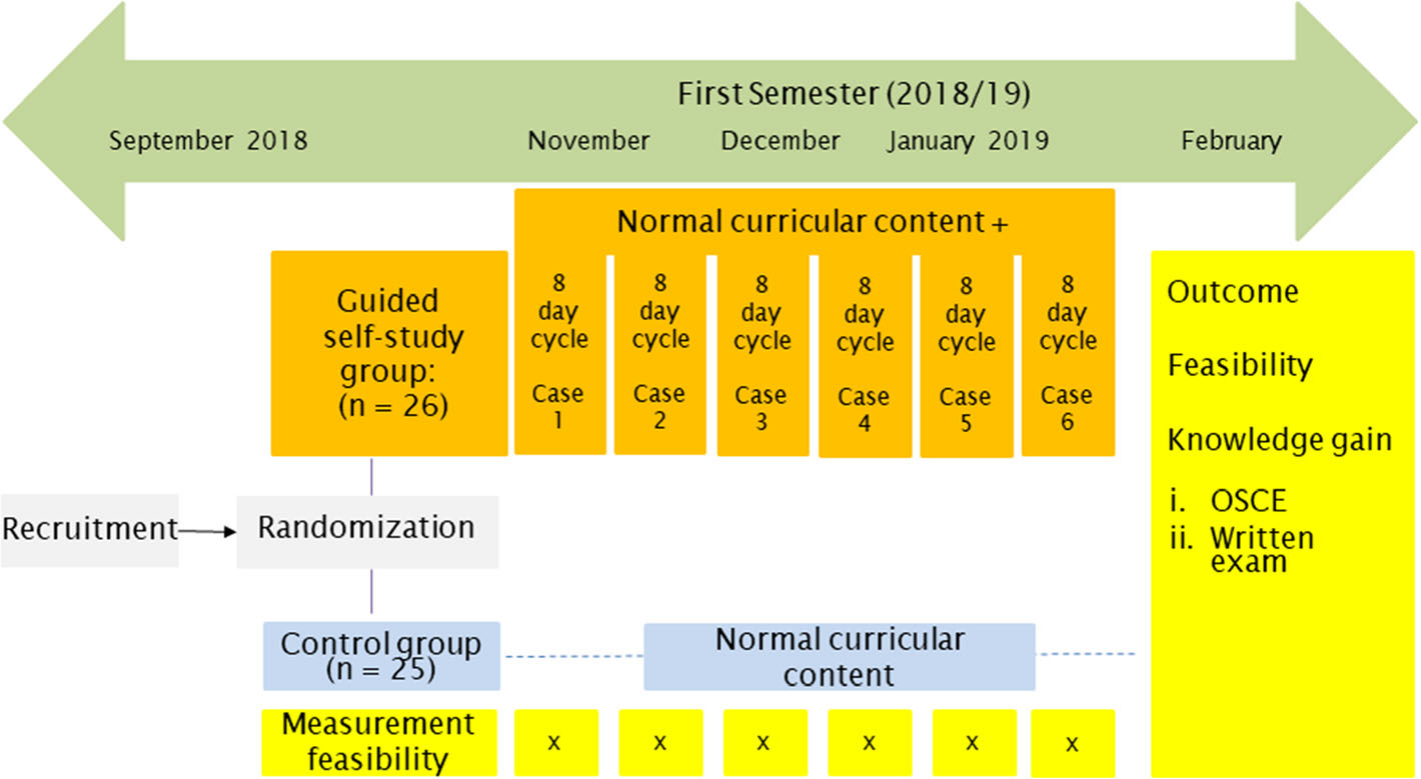
Overflow of the guided self-study

Figure 1 depicts the flow of this feasibility study design.

This cohort randomized feasibility higher education trial was designed as a prospective, single-center, two-arm design. This study was conducted at the BFH Department of Health Professions, Division of Physiotherapy. Undergraduate physiotherapy students (*n* = 51) from the first semester were invited to participate.

This manuscript was written following the CONSORT 2010 checklist [17]. The ethics committee of the Canton of Bern (Switzerland) approved this feasibility study was registered at the Registry of Efficacy and Effectiveness Studies (REES: ID: #1726.1). Participants and recruitment.

Inclusion criteria for the current higher education feasibility study were young healthy physiotherapy students of the undergraduate physiotherapy degree course 2018 from the first semester of the BFH in the German speaking part of Switzerland. Exclusion criteria were physiotherapy students of the undergraduate physiotherapy course 2017 who needed to repeat the first semester, students from other BFH degree courses or from other institutions.

In Switzerland, suitability of applicants for the undergraduate physiotherapy program is examined in a two-stage test procedure. The best 51 candidates will be selected for the fulltime study course. Hence, a high degree of homogeneity concerning their school-leaving qualifications and previous knowledge can be assumed.

Recruitment of physiotherapy students of the undergraduate physiotherapy course 18 for this present feasibility study was done by means of an oral information session with distribution of declarations of consent. The potential study participants were given 2 weeks’ time to decide for or against voluntary study participation. All participants provided written informed consent.

### Randomization

Randomization of undergraduate physiotherapy students into groups is a standard procedure at BFH to keep the group size for practical lessons small and to promote group learning. Randomization was computer generated by an independent researcher and resulted in a tutor-G-SS group (*n* = 26) and a control group (CG; *n* = 25).

### Intervention

The process was based on the previous published Rogan’s recommendations to develop G-SS [9]: a total of six G-SS periods were scheduled for the G-SS group between the start of November 2018 and mid of January 2019. The G-SS period consisted of an 8-day cycle in which a clinical case was processed. In total six weeks were scheduled, whereby one clinical case peer week was processed.

Clinical cases were used for the G-SS sessions that aligned to the module contents of the undergraduate physiotherapy degree program and which were not targeted to the semester exam. Table 1 gives an overview of the contents of the clinical cases in the tutor-G-SS sessions.

**Table 1:** Overview of the G-SS clinical cases proposed in eight-day cycle procedure

G-SS period	Clinical Case	Learning objective
1	Thoracic massage of an elderly person after heart surgery	1. To perform massage techniques on two different positions 2. To develop a massage checklist
2	Colleague with a muscle stiffness in the region of the hamstring after Squash	1. To develop an examination protocol 2. To explain a physiological reflex model of muscle stiffness
3	Gait analysis of an elderly person and younger person	1. To develop a gait analysis checklist 2. To develop an examination protocol for gait analysis
4	Measurement of body joint angles with goniometer and mobile-phone-based apps	1. Explaining the differences between the neutral-zero measurement method and Apps applications 2. To develop a checklist for traditional joint angle measurement for hip and knee joint mobility.
5	Passive and active joint examination, translational joint examination and tests for muscle flexibility and muscle strength of the pelvis-hip-region	1, To perform a specific examination of the hip region in a time frame of 8 min
6	Football player with knee pain with a pain area around the adductor tubercle	1. Hypothesis-deductive approach of an examination of the lower extremity

**Fig. 2:**
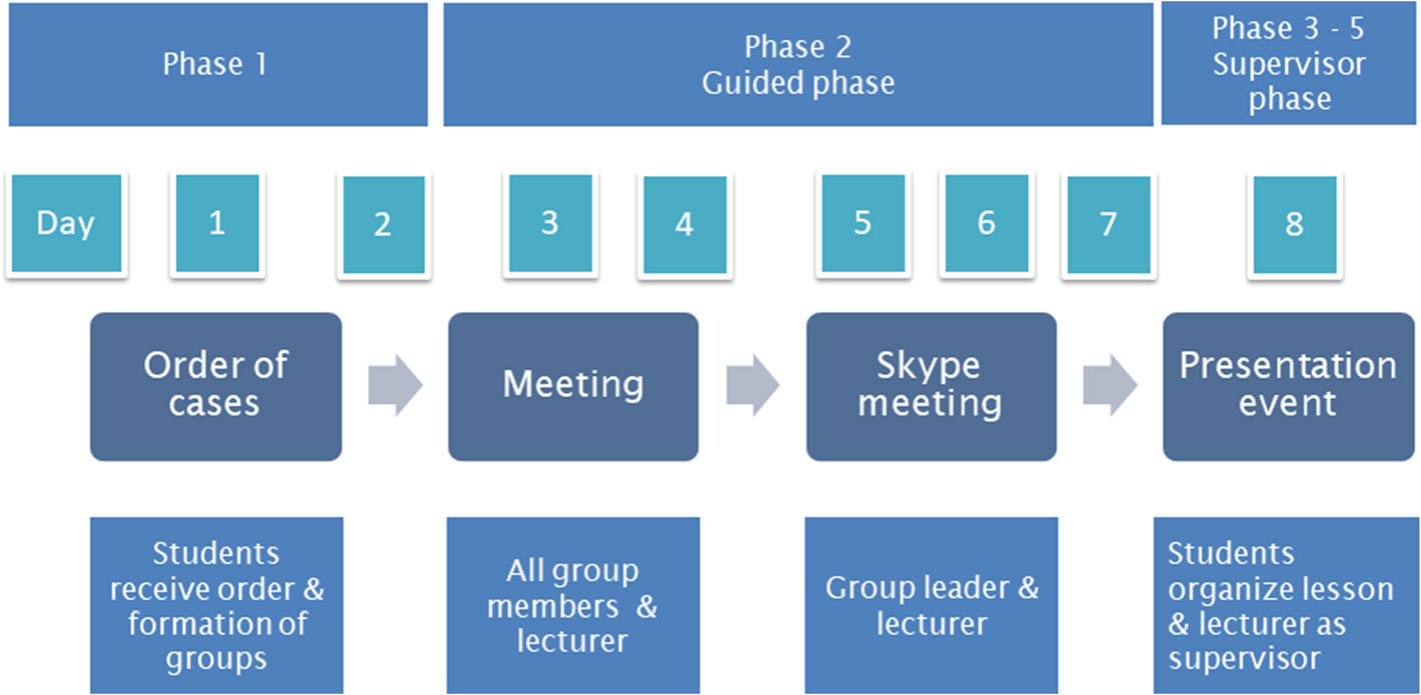
Eight-day cycle of the guided self-study

Figure 2 depicts the flow of the eight-day cycle intervention.

On day 1, 1 week prior to the G-SS session, the students of the G-SS group were informed about the learning goals and received the clinical case description and corresponding tasks via email (phase one). From day 2 to 7, students could choose between a tutor-supported approach or not (phase two). On day 8, the G-SS groups presented results of their work to the tutor and to their peers (phase three). Students carried out an oral reflection on their work (phase four). At the end of day 8, the tutor moderated an in-class plenary session including feedback (phase five). The duration time of each G-SS session was 90 min. The tutor was a higher education lecturer from BFH with 19 years of teaching experiences.

### Control group

Students are self-reliant in F-SS and performed F-SS as planned in the traditional curriculum of the bachelor’s degree course.

### Outcomes

#### Primary outcome: feasibility

The primary outcome measure of feasibility was the fidelity of implementation [18, 19]. The fidelity of implementation was measured as follows: *i)* Exposure was measured as 1. the total number of the conducted G-SS sessions and 2. the duration of each G-SS session in minutes, *ii)* Students responsiveness was documented by the tutors in the attendance list after each G-SS session (Phase 3–5) and by a post-study oral group interview survey. An adequate responsiveness to the protocol was defined that every student of the G-SS group should have attended five of six G-SS session, with 83% of students consenting. *iii)* Program differentiation was evaluated in the course of the program conception, we examined which program-related and competing contents could be observed in the G-SS cases and the curriculum.

#### Secondary outcome: impact of intervention

The Kirkpatrick’s model [15, 20, 21] was used to evaluate students’ learning outcomes. Kirkpatrick’s model is based on four levels, 1) reaction, 2) learning, 3) behavioral changes and 4) organizational performance. It is usually not possible to measure all four levels at once [22]. Therefore, Level 2 has been implemented in this study. Students were assessed twice per semester for written (multiple choice; MC) and practical (objective structured clinical examination; OSCE) competences. These two exams were part of the undergraduate study of physiotherapy. The total score for the MC exam was 94 points. The OSCE consisted of eight stations with a total score of 48 points (i.e.6 points per OSCE station). Students passed if a score of 60% for MC and OSCE was reached.

### Statistics

To analyze the effect of G-SS on the final grades of the written exam and OSCE (dependent variables) the number of attendances of each student on the presentation day of the G-SS was used as an independent variable.

For the secondary outcome measures, descriptive statistics were conducted and presented as means with corresponding standard deviations (SD). To guarantee that the randomization remains unbroken, an intention-to-treat analysis (ITT) was performed, where missing data were replaced by mean values of the group to which subjects were originally allocated [23]. The student’s t-test was applied to determine differences in the exam results between the two groups after the first semester. All calculations were performed using Statistical Package for Social Sciences (SPSS) version 25.0 (IBM Corp. Released 2017. IBM SPSS Statistics for Windows, Version 25.0. Armonk, NY: IBM Corp.)

## Results

This manuscript reports data for 51 undergraduate physiotherapy students from the BFH. All students volunteered in this higher education feasibility study.

### Primary outcome

#### Feasibility

Fidelity of implementation of exposure of the G-SS was that all planned six G-SS sessions were carry out in the planned time of 90 min at day 8.

Fidelity of implementation of students’ responsiveness to the G-SS program was 61%. All students participated in at least three G-SS sessions at day 8, while 16 students (61.5%) participated in four sessions at day 8, six students (23.01%) participated in five sessions at day 8, and two students (0.07%) participated in all six G-SS sessions at day 8. Around 90% of the students attended the first (November 2018), the fifth and sixth G-SS session (both January 2019) at day 8. The 83% target of five attendances per student was not achieved.

Post-study interviews revealed the following findings: The responses referred to a clear description of the clinical cases, while the learning goals were well aligned with the content of the curriculum. However, the time frame for the preparation of the clinical cases seemed not optimally embedded in the academic timetable. G-SS was scheduled into the timetable, where the student’s workload was very high. The timetable was scheduled with lessons until 5 p.m. Hence, from the student’s perspective, there was no adequate time to work on the tasks.

Fidelity of implementation of program differentiation determined no duplication of contents from the regular program schedule in the evaluation.

#### Secondary outcome

Table 2 shows the OSCE total score for the entire exam and the total score for the MC exam if students attended five times (phase 3 to 5) and if students attended six times (phase 3 to 5) the G-SS session. An ITT was performed for all OSCE and MC values. The total score for the MC and those for the OSCE total scores, and the total scores at each OSCE station in the first semester were used as outcome variables.

**Table 2:** Overview of MC total score, OSCE total scores and each OSCE stations total score in mean and standard deviation (SD). ITT results are presented

	G-SS group (*n* = 26) Mean (SD) (5 attended G-SS sessions at day 8)	G-SS group (n = 26) Mean (SD) (6 attended G-SS sessions at day 8)	CG (*n* = 25) Mean (SD)	P (5 attended G-SS sessions at day 8)	P (6 attended G-SS sessions at day 8)
OSCE total	40.55 (± 0.44)	41.96 (± 0.19)	39.73 (±3.34)	0.114	0.003*
MC	62.35 (± 3.00)	66.10 (± 2.47)	60.74 (± 7.73)	0.259	0.003*
OSCE station 1	5.30 (± 0.32)	5.18 (± 0.23)	5.36 (± 0.56)	0.649	0.001*
OSCE station 2	5.23 (± 0.23)	5.10 (± 0.12)	4.73 (± 0.89)	0.007*	0.039*
OSCE station 3	5.43 (± 0.23)	5.30 (± 0.42)	4.99 (± 0.93)	0.029*	0.103
OSCE station 4	4.94 (± 0.12)	5.57 (± 0.27)	4.98 (± 0.53)	0.715	0.001*
OSCE station 5	5.04 (± 0.23)	5.25 (± 0.07)	4.93 (± 0.59)	0.368	0.008*
OSCE station 6	4.50 (± 0.30)	4.78 (± 0.21)	4.75 (± 0.59)	0.928	0.797
OSCE station 7	5.35 (± 0.22)	5.33 (± 0.05)	5.18 (± 0.66)	0.238	0.262
OSCE station 8	4.56 (± 0.34)	5.12 (± 0.21)	4.71 (± 0.62)	0.274	0.001*

* p = < 0.05 (student’s t-test)

## Discussion

According the recommendation of the Rektorenkonferenz der Fachhochschulen der Schweiz [8], the Department of Health Professions at the BFH has the responsibility to design a student-centered curriculum with self-study periods. Implementing G-SS in the physiotherapy undergraduate curriculum could improve teaching and could foster students-centered learning approach. This feasibility study evaluated the fidelity of implementation (whether G-SS was implemented at all) to explain succeed and fail of G-SS implemented in comparison with the original program design. In addition, the changes in knowledge and skills in undergraduate physiotherapy students at the BFH were assessed.

The research question “it is possible to develop a definitive study design that will evaluate knowledge changes in undergraduate physiotherapy students?” could be answered as follows: this present study design is not appropriate to assess knowledge changes in undergraduate physiotherapy students. We were able to demonstrate that G-SS as originally planned and scheduled in the academic timetable is not feasible. A modification of the study design must be carried out.

Regarding the fidelity of implementation of exposure, it could be demonstrated that all six G-SS sessions at day 8 were performed in the planned time of 90 min. In terms of exposure, it can be assumed that program are less effective if the target group does not receive the number of intended interventions [24].

Only six students (23.1%) instead of the expected 83% participated in five G-SS sessions at day 8. The first session was very well frequented, because this session was scheduled in a time period with normal workload of < 40 h. The fifth and sixth GSS sessions (day 8) was used by the students as exam preparation units > 45 h. The learning workload increased in December of the circumstances that students must additionally write a project thesis. Another reason was that the timetable in November and December 2018 were designed with a higher learning workload to allow sufficient learning preparation time in January 2019 for the exam in February 2019. The scheduling of the G-SS in the timetable was mentioned by the students as the reason for the low acceptance. This is in line with the findings of the literature.

Newble and Entwistle [25] postulated that student learning is influenced by individual characteristics and by external circumstances. Exam and learning workload of the curriculum are examples of such external learning circumstances. Exams can be a strong stimulus for learning [26]. Curricular circumstances are further determinants of learning [25]. Workload was very high between November and December 2018 (> 45 h per week) while module exams were scheduled at the beginning of February 2019. It is known that curricula influence undergraduate students’ preference for learning environments [24]. The analysis showed that the quality of the G-SS cases was rated as good. The contents of the cases and goals of the cases were aligned with the curriculum. This promotes the willingness to accept the clinical cases and not to reject them in advance. The cases (Table 1) can be transferred in this form to the next study.

Based on the findings of this feasibility study, the following modifications must be considered for the upcoming study design. G-SS study should be scheduled in time periods where workload is in normal range (< 40 h per week). To avoid further increase of workload, such an intervention should not be planned on top of an existing curriculum, but preferably be integrated as a part of the curriculum. It must also be taken into account that the learning workload during the module is evenly distributed.

### Preliminary impact of the intervention

TCI and seminars are the most suitable to promote factual knowledge [24]. In order to develop practical skills, other learning methods must be offered [24]. This present study found that all students in the G-SS group passed both exams, while students in the CG (*n* = 4) failed for both exams. Moulaert et al. [27] explained that repeated practice of a skill is particularly important for the learning process. The ability to independently acquire knowledge and skills is an important prerequisite for clinical work [26]. G-SS meets this requirement because the preparation of the clinical case, the presentation and the student’s feedback as reflection process were included. Results of the present study suggested that students attending the G-SS sessions, as a form of SDL, six times, showed beneficial changes of knowledge and skills.

Positive effects of SDL on knowledge was also found by Murad et al. [28]. These authors conducted a meta-analysis including 50 articles totaling 8000 health professionals’ students to illustrate the effectiveness of the SDL versus traditional teaching methods on knowledge domain. SDL showed significant gain in factual knowledge. The future study should be structured according the SDL principle. The university lecturer should guide the students in which meetings are offered. Furthermore, students should be involved in their learning process. In the preliminary stage, the learning methods must be clarified. The first three cycles of the G-SS program, the scheduled meetings with the university lecturers (tutors) should be declared as mandatory meeting. The remaining meetings during the last three cycles of the G-SS program can be declared as voluntary meetings.

### Study limitations

A possible weakness of this feasibility study was that confounding variables such as communication, motivation or self-regulatory skills were not assessed. Despite the randomization, this may have influenced the results. Further studies should measure these variables prior the baseline. However, in education research the use of clinical study designs has been questioned. Indeed, the concept that learners can be “exposed” to learning or education and that a specific number of sessions would be associated with learning achievement as outcome may be problematic. Also, a more sophisticated RCT with mor advanced statistical analyses will not necessarily lead to better study results. Experts in the field of education research consider non-RCT methods not as inferior to RCT.

Another limitation was the measurement of the number of fidelities of implementation criteria. Dusenbury et al. [29] postulated five fidelity of implementation criteria. 1. Adherence: were the components of the intervention being delivered as designed. 2. Exposure: number, length, or frequency of sessions implemented. 3. Quality of delivery: the way in which the program was delivered using the techniques, processes, or methods prescribed. 4. Participant responsiveness: participants engagement by and involved in the activities and content of the program. 5. Program differentiation: if there are critical characteristics that distinguish the program from the comparison condition, are they present or not during implementation. In this present study, qualitative methods were used. However, the power improves if statistical quantitative measure of fidelity will be carried out. Further studies should use qualitative and quantitative methods.

Additionally, three of the five criteria have been measured. The next study should take into account to evaluate all five criteria of fidelity of implementation. In addition, more qualitative related questions may be asked such as satisfaction with the G-SS sessions or if G-SS increased learning motivation in students.

## Conclusion

This study presents the feasibility of a G-SS program based on the learning method SDL that has been developed for first semester undergraduate physiotherapy students at the BFH in Switzerland. Findings from the results suggest that the developed study design was not feasible in its current form, and the study design must be modified for future studies. All six G-SS session were carried out as scheduled, student’s attendance to G-SS sessions was low with 23.02% instead of the expected 83%. The scheduling of the G-SS sessions into the period of a high workload was considered as the main reason for this low attendance. Future studies must consider the workload of the timetable when planning the G-SS.

G-SS has the potential to beneficially effect changes in knowledge and skills when students prepare and present all clinical cases and give feedback as reflection process at the end of each of six sessions.

## Data Availability

The datasets used and/or analyzed during the current study is available from the corresponding author on reasonable request.

## Abbreviations

BFH: Bern University of Applied Sciences
CG: control group
ECTS: European Credit Transfer System
GSG: guided self-study group
ITT: intention-to-treat-analysis
MC: multiple choice
OSCE: objective structured clinical examination
SD: standard deviation
SPSS: Statistical Package for Social Sciences
